# Prevalence and Clinical Characteristics of Patients with Torsades de Pointes Complicating Acquired Atrioventricular Block

**DOI:** 10.3390/jcm12031067

**Published:** 2023-01-30

**Authors:** Sok-Sithikun Bun, Nathan Heme, Florian Asarisi, Fabien Squara, Didier Scarlatti, Pamela Moceri, Emile Ferrari

**Affiliations:** Faculty of Medicine, Pasteur University Hospital, 06000 Nice, France

**Keywords:** atrioventricular block, torsades de pointes, QT interval

## Abstract

Background: Female gender, degree of QT prolongation, and genetic susceptibility are known risk factors for developing torsades de pointes (TdP) during high-grade atrioventricular block (HG-AVB). Our objective was to analyze the prevalence and clinical characteristics of patients presenting with TdP and AVB (TdP [+]) in comparison with non-TdP patients with AVB (TdP [−]). Methods: All the ECGs from patients prospectively admitted for AVB (2 to 1, HG, and complete) at the University Hospital of Nice were analyzed. Automated corrected QT (QTc), manual measurements of QT and JT intervals, and Tpeak-to-end were performed at the time of the most severe bradycardia. Results: From September 2020 to November 2021, 100 patients were admitted for HG-AVB. Among them, 17 patients with TdP were identified (8 men; 81 ± 10 years). No differences could be identified concerning automated QTc, manual QTc (Bazett correction), baseline QRS width, or mean left ventricular ejection fraction between the two groups. Potassium serum level on admission and mean number of QT-prolonging drugs per patient were not significantly different between the two groups, respectively: 4.34 ± 0.5 mmol/L in TdP [+] versus 4.52 ± 0.6 mmol/L (*p* = 0.33); and 0.6 ± 0.7 in TdP [+] versus 0.3 ± 0.5 (*p* = 0.15). In contrast, manual QTc_FR_ (Fridericia correction), JT (Fridericia correction), Tpeak-to-end, and Tpe/QT ratio were significantly increased in the TdP [+] group, respectively: 486 ± 70 ms versus 456 ± 53 ms (*p* = 0.04); 433 ± 98 ms versus 381 ± 80 ms (*p* = 0.02); 153 ± 57 ms versus 110 ± 40 ms (*p* < 0.001); and 0.27 ± 0.08 versus 0.22 ± 0.06 (*p* < 0.001). Conclusions: The incidence of TdP complicating acquired AVB was 17%. Longer QTc_FR_, JT, and Tpeak-to-end were significantly increased in the case of TdP but also in the presence of permanent AVB during the hospitalization.

## 1. Introduction

Torsades de pointes (TdP) are a specific form of polymorphic ventricular tachycardia that is preceded by QT interval prolongation and occurs in a variety of conditions [1]. TdP is a known but uncommon complication of atrioventricular block (AVB) and may recur in some patients, even after permanent cardiac pacing [2]. Female gender [3], degree of QT prolongation [4], and genetic susceptibility [5] are all known risk factors for developing TdP during acquired AVB. While electrocardiographic (ECG) parameters predicting the occurrence of TdP have been well described, little is known about the clinical characteristics of the patients presenting with this life-threatening complication. Identifying predisposing factors for developing TdP may help in better discriminating a higher-risk group and avoiding any recurrence of TdP.

Our objective was to analyze the prevalence, clinical, and ECG characteristics of patients prospectively admitted for AVB and TdP (TdP [+] group) in comparison with non-TdP patients (TdP [−] group).

## 2. Materials and Methods

All the patients admitted at the University Hospital of Nice for acquired AVB were prospectively included in this study, and their electrocardiograms (ECGs) were systematically collected. The cohort included patients presenting with 2 to 1, high grade (HG), and complete AVB leading to bradycardia that was severe enough to justify hospital admission. The patients with the following forms of AVB were excluded from this study: AVB in the setting of acute myocardial infarction, drug toxicity, or vagally mediated episodes. All the patients underwent continuous cardiac monitoring during their hospitalization stay, until pacemaker implantation (if needed). The ECG tracings (12 lead surface and telemonitoring during hospitalization) were reviewed by one experienced electrophysiologist (S.-S.B.). AVB was classified as permanent in this study if complete AVB was observed throughout the hospitalization stay during cardiac monitoring, or intermittent if complete AVB was observed in alternation with 1:1 conduction (eventually facilitated by isoproterenol infusion).

**Figure 1:**
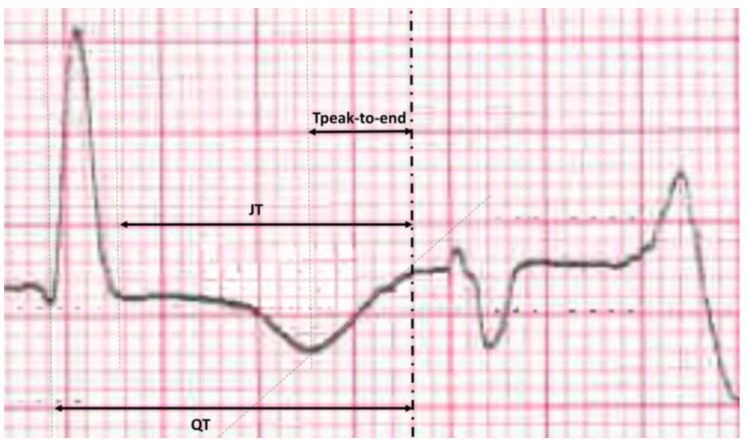
Method of manual measurement for QT, JT, and Tpeak-to-end intervals at the moment of most severe bradycardia.

TdP was defined as polymorphic ventricular tachycardia (faster than 120 beats per minute and at least three consecutive QRS complexes originating from the ventricles), with axis rotation and variable QRS complex amplitudes. Patients were assigned to the TdP [+] group if more than 10 TdP beats were recorded during telemetry. The clinical and electrocardiographic characteristics of the patients were analyzed, as well as the number of QT prolonging agents and potassium serum level on admission. ECGs were recorded at a gain of 10 mm/mV and a paper speed of 25 mm/s. Automated corrected QT intervals (QTc_AUTO_) were collected, and manual measurements of the following intervals were performed at the time of the most severe bradycardia: RR, QT, JT, and Tpeak-to-end intervals (Figure 1). For manual measurements, QT intervals were measured from the onset of the QRS complex to the end of the T wave, which was defined as the point of its merger with the isoelectric line. QTc is the QT for the heart rate using the Bazett formula (QTc = QT/square root of RR) [6], whereas QTc_FR_ uses the Fridericia heart rate correction formula [7]. T peak-to-end was measured from the summit of the T wave to the end of the QT interval. These intervals were determined as a mean value derived from three consecutive cardiac cycles. Both QT, JT, and Tpeak-to-end intervals were measured in the ECG leads with the longest value. Corrected (JTc) is the JT interval for the heart rate with the Fridericia formula correction.

This study was approved by the Institutional Review Board. According to our institutional guidelines, all patients gave written informed consent for the pacemaker implant (if needed).

### Statistical Analysis

The statistical analysis was performed using Excel (San Diego, CA, USA). Categorical variables are described as numbers and percentages. Continuous variables are described as mean ± SD for normal distributions or median for a range for non-normal distributed. Between-group differences in categorical variables were compared using a chi-squared test. Differences between continuous variables were compared using a Student’s t test. To test whether different variables were influencing the occurrence of TdP, a binary logistic regression was performed with TdP as the dependent variable and other variables as covariates.

## 3. Results

**Figure 2:**
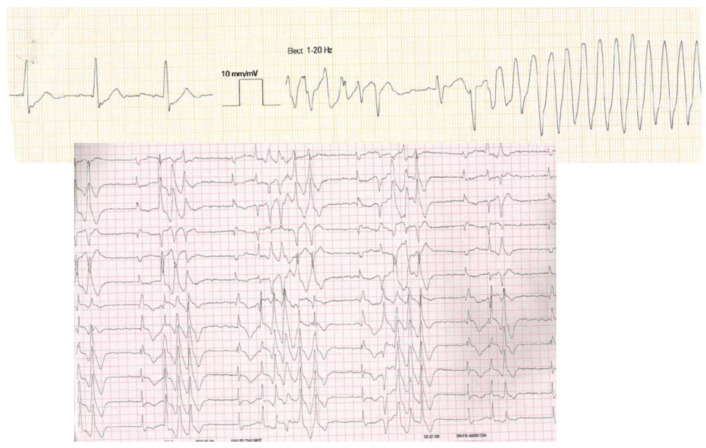
Twelve-lead ECG from an 82-year-old female patient admitted to the emergency department for syncope with brain trauma. The initial ECG shows atrial fibrillation with complete atrioventricular block and ventricular escape rhythm (with right bundle branch block morphology) and salvos of torsades de pointes. The initial episode required urgent electrical cardioversion.

From September 2020 to November 2021, 100 patients were admitted for HG or complete AVB in our institution. Among them, 17 patients (17%) with TdP were identified (8 men; 81 ± 9 years). A total of 13 out of 17 patients presented with several episodes of TdP (76%). An example is shown in Figure 2. Arterial hypertension was present in 10/17 (59%), with a mean left ventricular ejection fraction = 57 ± 6%. A comparison with the other patients admitted with complete AVB but without TdP is shown in Table 1. No clinical characteristics could distinguish patients presenting with TdP complicating AVB, in comparison with other patients admitted without TdP [−], except concerning the intermittent nature of AVB. Permanent AVB during the hospitalization stay was statistically more prevalent in TdP [+] patients (14 out of 17, 83%) versus 43/83 (52%) in TdP [−], *p* = 0.02. Two patients (12%) in TdP [+] elicited a pause-dependent (phase 4) AVB mechanism versus 12 (14%) in TdP [−], *p* = 0.77 [8].

**Figure 3:**
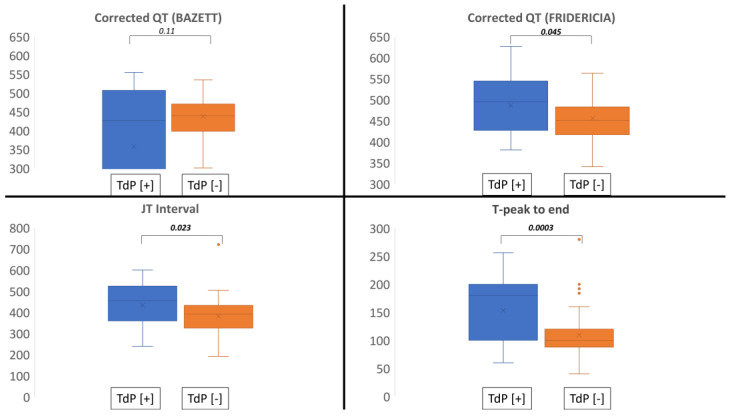
Box plots of corrected QT with Bazett’s formula (**upper left**), Fridericia’s formula (**upper right**), the JTc interval (**lower left**), and Tpeak-to-end (**lower right**) between the two groups. Bold, significant on statistical analysis.

No differences could be identified between the two groups concerning QT_cAUTO_, baseline QRS width, or mean left ventricular ejection fraction. Potassium serum level on admission and mean number of QT-prolonging drugs (Supplementary Materials) per patient were not significantly different between the two groups, respectively: 4.34 ± 0.5 mmol/L in group TdP [+] versus 4.53 ± 0.6 mmol/L (*p* = 0.13) and 0.57 ± 0.7 in group TdP [+] versus 0.34 ± 0.5 (*p* = 0.13). QTc, QT_cFR_, the JTc interval, and Tpeak-to-end were significantly prolonged in the TdP group in comparison with the TdP [-] group (Figure 3). Univariable analysis with logistic regression was statistically significant for the presence of permanent AVB during hospitalization, QT_cFR_, Tpeak-to-end, and Tpeak-to-end/QT (Table 2). QTc was an independent predictor of TdP in a logistic regression model using age, gender, the presence of permanent AVB, the R-R interval, and Tpeak-to-end as covariates (*p* = 0.04).

## 4. Discussion

### 4.1. Prevalence of TdP in Patients with Atrioventricular Block

Available data are sparse concerning the prevalence of TdP in AVB patients, reaching 11% in older reports [2]. In this initial report describing ventricular tachycardia-ventricular fibrillation (and not TdP), the proportion could rise to 30% in the case of permanent AVB, as compared to partial AVB (4%). Later, Cho et al. reported a prevalence of TdP of around 2.2% in a recent retrospective study including 898 AVB patients from three tertiary hospitals over a 20 year period [9]. In this retrospective study, the authors excluded at least one third of the initial cohort of patients with TdP for various reasons: drug-induced QT prolongation, electrolyte disturbance, TdP during pacing rhythm, or absence of baseline ECG. Our study reports a prevalence of TdP (17%) complicating AVB in a tertiary medium- to high-volume center. This is in line with the most recent study, which reported an incidence rate of 25% of TdP in their retrospective cohort (100 patients), 12.6% of TdP in a cohort of 87 patients admitted with AVB in the prospective group, and a final prevalence of 18.8% among a pooled number of 250 patients from three distinct cohorts [4]. These variations may also be attributed to the classification of the patients: QT-prolonging agents were excluded in previous reports, whereas patients taking QT-prolonging drugs were included in our analysis.

### 4.2. Clinical Characteristics of Patients with TdP and Atrioventricular Block

As reported in early observations, TdP may be associated with Adams–Stokes seizures in addition to syncopal episodes related to the asystole itself [2]. This may produce another aggravating situation for trauma to occur at initial presentation. The observation of TdP during AVB may suggest the possibility of prompt management of AVB using a temporary pacing lead or externalized reusable permanent pacemaker (in the case of drug toxicity inducing QT prolongation, for example, allowing time for drug wash-out) [10,11]. There are no current recommendations for pacing programming after implantation in patients with TdP during AVB at presentation. It has been reported that TdP may recur in these patients even after permanent cardiac pacing, with the necessity for increasing the lower pacing rate from 60 to 80 or 90 beats/min, and eventually, adding a β-blocking agent. Identifying predisposing factors for developing TdP may help in better discriminating a higher-risk group and avoiding any recurrence of TdP, for instance, by anticipating pacemaker programming.

Another concern should be the development of pacing-induced cardiomyopathy and the likelihood of ventricular arrhythmia recurrence in patients with initial TdP on admission [12]. It is also not known whether conduction system pacing could represent a better strategy in this subgroup of patients with TdP complicating AVB by avoiding any recurrence during long-term pacing [13].

The majority of studies published in the literature focused on ECG features that could predict the occurrence of TdP during acquired AVB, but few studies exist on the clinical characteristics associated with TdP. In our study, no significant differences concerning the clinical features could be found between the two groups. Other parameters such as serum potassium level, renal function, or the number of QT-prolonging agents at the time of TdP were also not different between the two groups. Cho et al. did not find any difference in clinical data (hypertension or diabetes mellitus) in the TdP patients when compared to the control group [9]. In our study, there were more syncopal episodes in the TdP [+] group, but without reaching statistical significance (*p* = 0.11). Finally, the most discriminant factor was the presence of permanent AVB at presentation, which was significantly increased in the TdP [+] group as compared to the TdP [−] group (*p* = 0.02). In previous studies, female gender was found to be an independent risk factor for TdP occurrence; this was not the case in our study [4].

### 4.3. ECG Predictors of TdP in Atrioventricular Block Patients

Different QTc cut-off values (that predicted the occurrence of TdP) have been reported in the literature (Table 3), ranging from 440 to 565 ms. This wide range of values may be explained by several factors. The method of QT measurement may vary from one study to another. For instance, the correction of the QT interval using the Bazett formula was the most frequently used correction in the majority of the studies concerning TdP complicating AVB. Noteworthily, limitations of QTc with the Bazett formula at slow rates have also been demonstrated, with higher performance of the Fridericia formula during bradycardia [14,15]. The measurements were usually performed by a single operator, except in the study by Cho et al., with very low inter-observer variability. In our study, one electrophysiologist performed the QT measurements to limit inter-observer variability, but QT automatic measurements were also performed [9]. Automatic QT measurements have been shown to be accurate with high agreement when compared to manual measurements [16,17]. In contrast with previous reports, QTc and QT_cAUTO_ values were not statistically different between the two groups in our study, but only QTc_FR_. Concerning the other ECG parameters, our findings were in total agreement with previously published studies, with JT interval and Tpeak-to-end values being significantly increased in the TdP [+] group [18]. The JT interval and the J-T_peak_ have been reported to be a more reliable marker of the torsadogenic risk in patients with long QT syndrome, ventricular conduction defect, or in receiving a QT prolongating agent [19,20,21].

### 4.4. Limitations

This is a monocentric study with a limited number of patients, but its prospective nature provided some new information about the expected prevalence of TdP complicating acquired AVB. Genetic testing was not systematically performed in our elderly TdP [+] group. Recent studies [5,25] found that the presence of a genetic disposition ranged from 17 to 36%. Chevalier et al. found 17% of HERG mutations only in patients with a QT interval above 600 ms and AVB. Of note, our TdP [+] patients were discharged with a list of medications that were contraindicated in the long QT syndrome.

## 5. Conclusions

The prevalence of TdP complicating acquired AVB was 17%. Longer QTc_FR_, increased JT interval, and Tpeak-to-end were electrocardiographic predictors of TdP. The presence of permanent AVB was more likely associated with TdP in comparison with intermittent AVB at initial presentation.

## Figures and Tables

**Table 1:** Characteristics of patients with TdP.

	TdP [+] (n = 17)	TdP [−] (n = 83)	*p*-Value
Age (years)	81 ± 10 (57–94)	83 ± 12 (14–102)	0.62
Men, n (%)	8 (47)	46 (55)	0.72
Arterial hypertension, n (%)	10 (59)	56 (67)	0.13
Syncope, n (%)	13 (76)	46 (55)	0.11
Permanent AVB, n (%)	14 (82)	43 (51)	0.02
LVEF (%)	57 ± 6	55 ± 8	0.73
eGFR (mL/min/1.73 m²)	54 ± 29	56 ± 25	0.77
Mean QRS duration (ms)	119 ± 24	118 ± 33	0.86
QT_cAUTO_ (ms)	479 ± 55	465 ± 51	0.31
R-R interval (ms)	1414 ± 429	1360 ± 408	0.62
Heart rate (beats per min)	46 ± 14	49 ± 17	0.58
QT_c_ interval (ms)	462 ± 64	438 ± 55	0.11
QT_cFR_ interval (ms)	486 ± 70	456 ± 53	0.045
JT_c_ interval (ms)	433 ± 98	381 ± 80	0.023
Tpeak-to-end interval (ms)	153 ± 57	110 ± 40	0.0003
Tpe/QT ratio	0.27 ± 0.08	0.22 ± 0.06	0.0005

**Table 2:** Summary of univariable and multivariable analysis (logistic regression) with TdP.

	Odds Ratio 95% Confidence Intervals	*p*-Value
Univariable analysis		
Age (for a 10 year increase)	1.11 (0.70–1.66)	0.62
Female gender	1.44 (0.50–4.19)	0.5
Permanent AVB †	4.44 (1.33–20.3)	0.02
Potassium serum level on admission (+0.1 mmol/L)	1.06 (0.96–1.19)	0.26
Number of QT prolonging drugs (+ 0.1)	0.93 (0.85–1.03)	0.17
Mean QRS duration (+10 ms)	1.02 (0.86–1.21)	0.82
R-R interval (+100 ms)	0.96 (0.85–1.08)	0.48
Heart rate (+10 bpm)	1.01 (0.98–1.05)	0.47
Corrected QT (Bazett) (+10 ms)	1.08 (0.99–1.18)	0.09
Corrected QT (Fridericia) (+10 ms)	1.09 (1.00–1.19)	0.05
Corrected JT (Fridericia) (+10 ms)	1.06 (0.99–1.15)	0.09
Tpeak-to-end (+10 ms)	1.20 (1.07–1.35)	<0.01
Tpeak-Tend/QT (+0.01)	1.14 (1.04–1.26)	<0.01
Multivariable analysis		
Age (for a 10 year increase)	0.99 (0.95–1.04)	0.92
Female gender	1.79 (0.64–5.22)	0.27
Permanent AVB †	2.51 (0.8–7.69)	0.71
R-R interval (+100 ms)	1.00 (0.99–1.00)	0.46
Corrected QT (Bazett) (+10 ms)	0.99 (0.98–0.99)	0.04
Tpeak-to-end (+10 ms)	0.98 (0.97–0.99)	<0.01

All electrocardiographic parameters refer to values during an atrioventricular block. † in comparison to intermittent AVB.

**Table 3:** Summary of published studies of ECG parameters in TdP patients during AVB.

Author	Design Number of TdP [+]/TdP [−]	Parameter	TdP [+]	TdP [−]
Strasberg B, 1986 [22]	Retrospective 9 vs. 12	QTc (ms)	510 ± 60 *	400 ± 40
Moroe K, 1988 [23]	Retrospective 6 vs. 9	QTc (ms)	580 ± 112 *	459 ± 37
Kurita T, 1992 [24]	Retrospective 6 vs. 8	QTc (ms)	585 ± 45 *	476 ± 58
Subbiah, 2010 [5]	Retrospective 11 vs. 33	QTc (ms) Tpeak-to-end (ms)	440 ± 93 * 147 ± 25 *	376 ± 40 94 ± 25
Cho MS, 2015 [9]	Retrospective 20 vs. 80	QT (ms) Tpeak-to-end (ms) Tpe/QT	716.4 ± 98.9 * 334.2 ± 59.1 * 0.49 ± 0.09 *	523.2 ± 91.3 144 ± 73.7 0.27 ± 0.11
Chorin, 2017 [4]	Retrospective and prospective 47 vs. 203	QTc (ms)	564 ± 81 *	422 ± 62
Our series, 2023	Prospective 17 vs. 83	QTc_FR_ (ms) Tpeak-to-end (ms) Tpe/QT JTc (ms)	486 ± 70 * 160 ± 57 * 0.29 ± 0.08 * 437 ± 89 *	456 ± 53 106 ± 35 0.21 ± 0.06 375 ± 71

* Variables are statistically significant in comparison with TdP [−] group.

## Data Availability

The datasets used and analyzed during the current study are available from the corresponding author on reasonable request.

## Supplementary Materials

The following supporting information can be downloaded at: https://www.mdpi.com/article/10.3390/jcm12031067/s1, Table S1: Usual treatments favoring QT prolongation in the TdP [+] group.
