# Supplementary material for: Prevalence and Clinical Characteristics of Patients with Torsades de Pointes Complicating Acquired Atrioventricular Block
Source: J Clin Med. 2023 Jan 30;12(3):1067. doi: 10.3390/jcm12031067 (PMC9917754; doi:10.3390/jcm12031067)
Supplement: Supplementary file 1 [file jcm-12-01067-s001.zip › jcm-2146301-supplementary.pdf]

Supplemental Table S1: Usual treatments favoring QT prolongation in the TdP [+] group.

Patient	Age / Sex	Usual treatment identified and favoring QT prolongation
**1**	94 y / F	Furosemide
**2**	88 y / F	No
**3**	87 y / F	No
**4**	93 y / M	Furosemide
**5**	90 y / M	Amiodarone
**6**	63 y / M	No
**7**	76 y / M	Furosemide / Risperidone
**8**	79 y / F	No
**9**	81 y / M	Fluticasone and Vilanterol / Salbutamol
**10**	72 y / F	No
**11**	57 y / M	No
**12**	82 y / F	No
**13**	85 y / F	No
**14**	86 y / M	Levetiracetam
**15**	77 y / M	No
**16**	85 y / F	No
**17**	85 y / F	Hydrochlorothiazide
